# Single-Cell RNA Sequencing Reveals the Interaction of Injected ADSCs with Lung-Originated Cells in Mouse Pulmonary Fibrosis

**DOI:** 10.1155/2022/9483166

**Published:** 2022-04-11

**Authors:** Mamatali Rahman, Zhao-Yan Wang, Jun-Xiang Li, Hao-Wei Xu, Rui Wang, Qiong Wu

**Affiliations:** ^1^MOE Key Laboratory of Bioinformatics, Center for Synthetic and System Biology, Tsinghua University, Beijing 100084, China; ^2^School of Life Sciences, Tsinghua University, Beijing 100084, China; ^3^AGECODE R&D Center, Yangtze Delta Region Institute of Tsinghua University Zhejiang, Jiaxing, China; ^4^TUS-HARVEST Biotech. Co. Ltd, China

## Abstract

Pulmonary fibrosis (PF) is a severe chronic lung disease with little effective treatment options other than lung transplantation. Adipose-derived mesenchymal stem cells (ADSCs) have been shown to exert therapeutic effects on PF, but the underlying mechanisms remain to be further elucidated. Here, we show the interaction of ADSCs and lung-originated cells at the single-cell level, using bleomycin- (BLM-) induced mice PF model and green fluorescent protein– (GFP–) labeled mouse ADSCs. The intratracheally injected ADSCs were successfully recollected with flow cytometry and, together with lung-originated cells, were subjected to single-cell RNA sequencing (scRNA-seq). The ADSC treatment drastically changed the transcriptomic profile and composition of lung cells, especially macrophages. We explored the signal pathway interactions between ADSCs and lung-originated cells, showing potentially regulative pathways including NGR, ANNEXIN, HGF, and PERIOSTIN. Our data indicate that the injected ADSCs increased the number of *Trem2*^+^ antiinflammatory lung macrophages and lowered further inflammation and fibrosis in the lung. Our work realized the direct analysis of injected ADSCs to explore its *in vivo* interaction with the lung environment under PF and may provide critical information for future engineering of ADSCs to achieve better therapeutic effects in PF.

## 1. Introduction

Pulmonary fibrosis (PF) denotes a heterogeneous group of severe and lethal interstitial lung diseases, involving progressive lung remodeling, alveolar destruction, excessive pathological extracellular matrix (ECM) deposition, and scarring of the lungs [1–3]. The pathogenesis involves multiple biological pathways, including inflammatory response, apoptosis, oxidative stress, and epithelial to mesenchymal transition [4–8].

PF is traditionally considered to be an irreversible lung disease that is refractory to various treatments, resulting in high rates of mortality and morbidity [9]. Recently, the administration of mesenchymal stem cells (MSCs) has emerged as a promising therapy for PF [10, 11]. MSCs have been isolated from various tissues such as the bone marrow, adipose tissue, and the stroma of organs. They exhibit self-renewal ability and the potential to differentiate into a variety of cell lineages, including chondrocytes, osteoblasts, and adipocytes [12, 13]. And it is reported that MSCs attenuate fibrotic diseases including PF, by immunomodulation, anti-apoptosis, antiinflammation, and antifibrosis functions [14–18]. Among several sources of MSCs, ADSCs have the advantages of easy accessibility and relative abundance, which make a practical cell source for cell therapy [19, 20].

**Figure 1:**
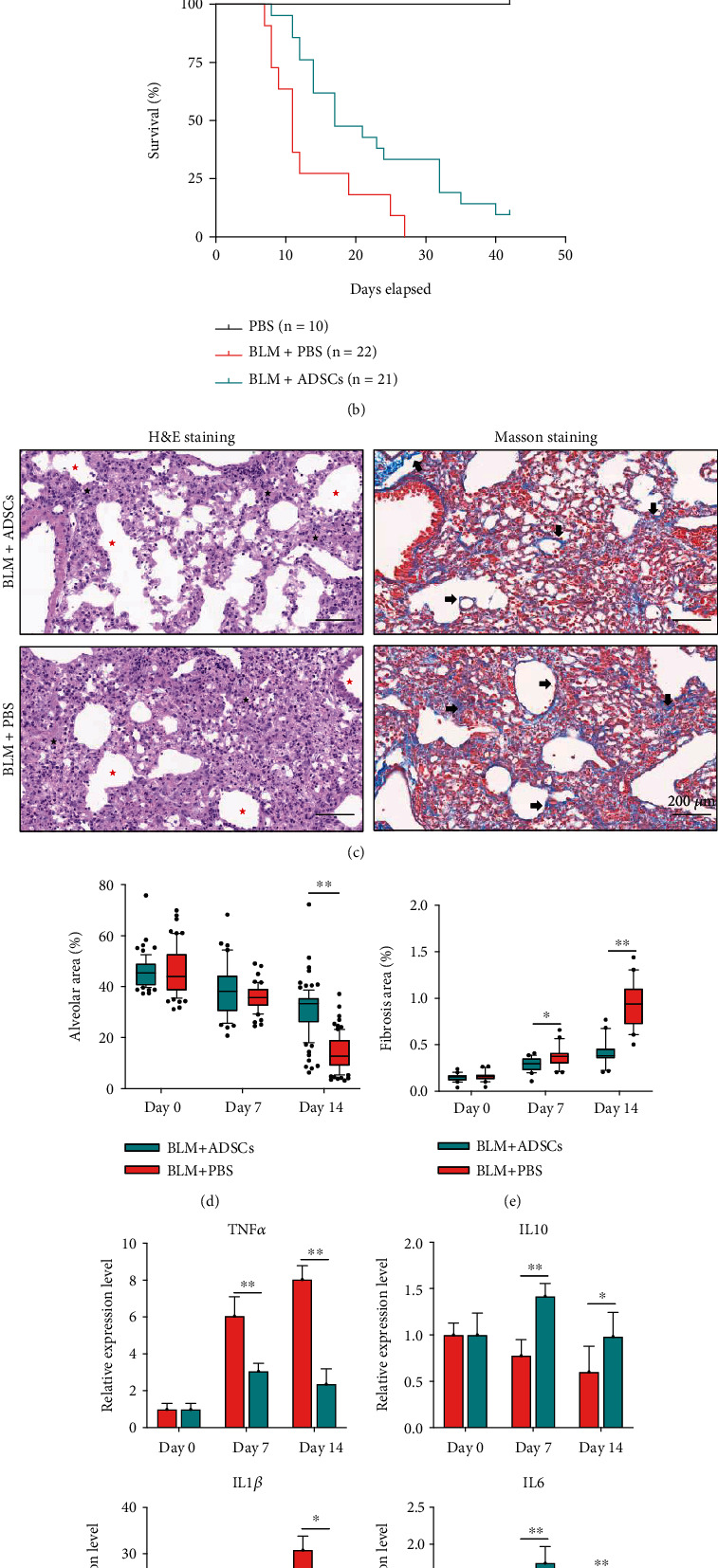
Intratracheal injection of ADSCs alleviated mouse pulmonary fibrosis. (a) The diagram of the study. (b) Kaplan-Meier survival curve for pulmonary fibrosis mice. The group was received PBS (*n* = 10, black line), the BLM + PBS group administered 3.5 mg/kg BLM in 50 ul PBS at day 0 (*n* = 21, red line), and BLM + ADSCs group received 5 × 10^5 cell in 50 ul PBS by intratracheally at day 3 post-BLM administration. (c) Masson's and H&E staining images of the lung tissues of mice on day 14, scale bar: 200 *μ*m. (d) Density of pulmonary fibrotic tissue (fibrosis area (black arrow)/total area × 100%) calculated on histological sections of Masson's trichrome staining. (e) Density of the pulmonary tissue (alveolar (red star) area ÷ parenchyma (black star) area × 100%) calculated on histological sections of H&E trichrome staining. (f). Relative expression of TNF-*α*, IL-1*β*, IL-10, and IL-6 mRNA in the pulmonary tissue of BLM + ADSC and BLM + PBS group (*n* = 3 each) determined by quantitative real-time polymerase chain reaction, on days 7 and 14. Data presented as mean ± SD with significance based on Student *t*-test. ∗*P* < 0.05; ∗∗*P* < 0.01.

The effects of ADSCs have been widely investigated using both *in vitro* cultures and *in vivo* models. But on one hand, direct recovery and analysis of injected ADSCs were not performed yet, likely due to in adaptation and apoptosis after entering the pathological environment [21]; on the other hand, current characterization of ADSC-treated BLM lung remains on the tissue (bulk) level, while scRNA-seq can provide a higher resolution of the therapeutic effect, as well as enabling cell-cell interaction inference from the transcriptomic data. Therefore, in this study, we recollected the injected allogenic mouse ADSCs from BLM-treated mouse lungs and subjected recollected ADSCs and lung-originated cells to scRNA-seq to analyze the crosstalk between injected ADSCs and the PF lung cells (Figure 1(a)) and to improve the understanding of the mechanisms behind the antifibrotic effect of ADSCs.

## 2. Materials and Methods

### 2.1. Animals and Experimental Design

All the animal experiments were conducted under the authorization of the Institutional Animal Care and Use Committee of Tsinghua University. 6-week-old male C57BL/6 J male mice, purchased from the Laboratory Animal Research Center of Tsinghua University, were housed in the specific pathogen-free experimental animal environment at the Laboratory Animal Research Center of Tsinghua University, 5~6 mice per cage with sterilized food and water ad libitum and a 12/12-hour light/dark cycle.

### 2.2. Isolation of Mouse ADSCs

6-week-old C57BL/6J male mouse with body-wide CAG-eGFP expression were obtained from Shanghai Model Organisms Center, Inc. (Shanghai, China). The ADSCs were obtained by euthanizing the mice, digesting their groin adipose tissue with collagenase type I (1.0 mg/ml, Sigma) and cultured in mouse ADSC basal medium (CYAGEN, China) in a humidified atmosphere comprising 5% carbon dioxide at 37 °C, according to a previous publication [22].

### 2.3. Construction of the BLM-Induced PF Mouse Model and Administration of ADSCs

The mice were randomly divided into three groups: (i) control group, (ii) BLM + PBS group, and (iii) BLM + ADSCs group.

Groups ii and iii mice were anesthetized with 2.5% tribromoethanol (0.8% NaCl, 1 mM Tris (pH 7.4), 0.25 mM EDTA (pH 7.4)) and administered 3.5 mg/kg bleomycin solution (Sigma B5507-15un) in 50 *μ*L of phosphate-buffered saline (PBS) intratracheally on day 0 using a 1 mL syringe with a 25G needle. Control animals were treated with PBS instead of BLM [23, 24].

The injection of ADSCs (at passages 3~6) was performed on day 3 post BLM treatment; 50 *μ*L of a cell suspension containing 1 × 10^7^ cells/mL in PBS (5 × 10^5^ cells/mouse) was injected intratracheally using a 1 mL disposable syringe with a 25G needle. For the control group and BLM + PBS group, 50 *μ*L PBS was injected instead. Before implantation, cells were washed three times to remove the culture medium. The duration of the study was set at four weeks, as the PF remodeling process in mice has been reported to be mostly complete (70~80%) within 3 weeks. All animals were weighed daily from day 0 to 20.

### 2.4. Histological Evaluation of Lung Damage and Collagen Deposition of BLM-Induced Lung Injury

To assess the development of PF, mice were assessed at day 7 and 14. For the lung tissue collection, mice were euthanized by CO_2_ inhalation. The chest cavity was exposed, and the lungs were perfused and washed with ice-cold PBS. Then, the lungs were fixed with 4% (v/v) paraformaldehyde in PBS and then processed into 5-*μ*m-thick paraffin sections mounted on glass slides.

For histological assessment of fibrosis accumulation, slices were stained with hematoxylin and eosin (H&E) or Masson's trichrome stain, respectively. Axio Scan.Z1 (Zeiss, Germany) was used to scan the samples with the 20× objective. The images were processed with ZEN 2.3. The lung tissue parenchyma density (alveolar area ÷ parenchyma area×100%) and fibrosis level (fibrosis area ÷ total area×100%) were calculated with the Image-Pro premier 3D 9.2.0 using random and non-overlapping fields for grading the lung injury and fibrosis level [25, 26].

### 2.5. Preparation of Single-Cell Suspensions

For the preparation of single-cell suspensions of mouse lung tissue, mice were sacrificed on the 4th day following ADSCs treatment (namely day 7), and the lungs were perfused with PBS. All samples of BLM + ADSCs and BLM + PBS groups were digested using the Lung Dissociation Kit, mouse (130-095-927, Miltenyi Biotec, Germany) according to the manufacturer's instructions. Cells were passed through 70 *μ*m and 40 *μ*m strainers to remove large pieces, after which the red blood cell lysis solution (Sigma Aldrich, St. Louis, MO, USA) was used to eliminate erythrocytes, followed by flow-assisted cell sorting of pulmonary cells (GFP^−^) and ADSCs (GFP^+^), respectively.

### 2.6. Preparation of the scRNA-Seq Library

We prepared the scRNA-seq libraries using the Chromium Next GEM Single Cell 3′ Reagent kit (version 3.1) (10× Genomics, USA). Libraries were sequenced on a HiSeq Xten VS NovaSeq 6000 sequencing platform (Illumina, USA), which generated 150 bp paired-end reads.

### 2.7. scRNA-Seq Data Processing

The scRNA-seq library sequences were demultiplexed, mapped to the mouse genome, and counted to generate a gene expression matrix using CellRanger (10× Genomics). Further data processing was done using the R packages Seurat3 (UMAP visualization, clustering, and gene expression plots), ReactomePA (REACTOME pathway enrichment analysis), and CellChat (cell-cell ligand-receptor interaction analysis).

### 2.8. Immunofluorescence Staining

For immunofluorescence staining of APOE and TREM2 in the (ii) BLM + PBS and (iii) BLM + ADSCs groups, mice were anesthetized and lungs were extracted on days 7 and 10. The left lung lobes were fixed with 4% (v/v) paraformaldehyde, paraffin-embedded and cut into 5-*μ*m-thick sections. Antibodies were purchased from Biolegend US and immunofluorescence were performed on the sections. The images were scanned using Axio Scan.Z1 and processed with ZEN 2.3.

### 2.9. Real-Time Quantitative PCR

To assess the gene expression levels, mice were euthanized for lung tissue collection and the total RNA was extracted using TRIZOL reagent (Invitrogen, USA) and reverse-transcribed into cDNA using the SuperScript II First-Strand Synthesis System Kit (GenStar, A215, China). The RT-PCR reactions were conducted using 2 × RealStar Green Fast Mixture (GenStar, A304, China) with 3 technical repeats. *GAPDH* served as the internal reference gene and data were analyzed using the 2^-△△CT^ method. The specific primers were as follows: *TNF-α* (sense, 5′-CAT CTT CTC AAA ATT CGA GTG ACA A-3′; anti-sense, 5′-TGG GAG TAG ACA AGG TAC AAC CC-3′), *IL-1β* (sense, 5′-AGG TCG CTC AGG GTC ACA AG-3′; anti-sense, 5′-GTG CTG CCT AAT GTC CCC TTG AAT C-3′), *IL-10* (sense, 5′-TAA GGC TGG CCA CAC TTG AG-3′; anti-sense, 5′-GTT TTC AGG GAT GAA GCG GC-3′), *IL-6* (sense, 5′-GGT ACA TCC TCG ACG GCA TCT-3′; anti-sense, 5′-GTG CCT CTT TGC TGC TTT CAC-3′), and *GAPDH* (sense, 5′-GTA GTT GAG GTC AAT GAA GGG-3′; anti-sense, 5′-TCG TCT CAT AGA CAA GAT GGT-3′).

### 2.10. Statistical Analysis

All the experiments were performed in at least three independent replicates, and data are presented as means ± SD and visualized using OriginPro or R software. The statistical significance of differences was assessed using two-way ANOVA followed by Tukey's multiple comparisons tests (∗*P* < 0.05; ∗∗*P* < 0.01.

## 3. Results and Discussion

### 3.1. ADSCs Mitigates PF by Decreasing ECM Deposition and Inflammation

In the BLM-induced PF mouse model, intratracheal injection of ADSCs significantly prolonged the survival of the mice in 45 days compared to the BLM + PBS group (Figure 1(b)). We quantitatively assessed the fibrosis and alveolar damage in the lungs by histological staining, which revealed a visible improvement in the lung texture in the ADSCs group on day 14, though not on day 7 (Figure 1(c)), which were further validated by quantification of the percentage of lung alveolar and fibrotic area (Figures 1(d) and 1(e)). The fibrotic area of the BLM + ADSCs group was 0.4% on day 14, compared to 1.1% in the BLM + PBS group (Figure 1(d)), while the lung alveolar area was 31.1 ± 3.1% and 14.3 ± 1.3% in the BLM + ADSCs and BLM + PBS group, respectively (control group was 46.1 ± 5.5%) (Figure 1(e)).

Similarly, quantitative real-time PCR showed that the proinflammatory cytokines tumor necrosis factor-alpha (TNF-*α*) and interleukin-1 beta (IL-1*β*) were significantly downregulated in the BLM + ADSCs group mouse lungs at days 7 and 14, while antiinflammatory cytokines IL-10 and IL-6 showed the opposite trend (Figure 1(f)). These observations demonstrate that ADSC treatment decreased lung ECM deposition, mitigated fibrosis, and inflammation in mouse PF model.

### 3.2. ADSCs Increased the Abundance of antiinflammatory *Trem2*^+^ Macrophages in BLM-Treated Lung

**Figure 2:**
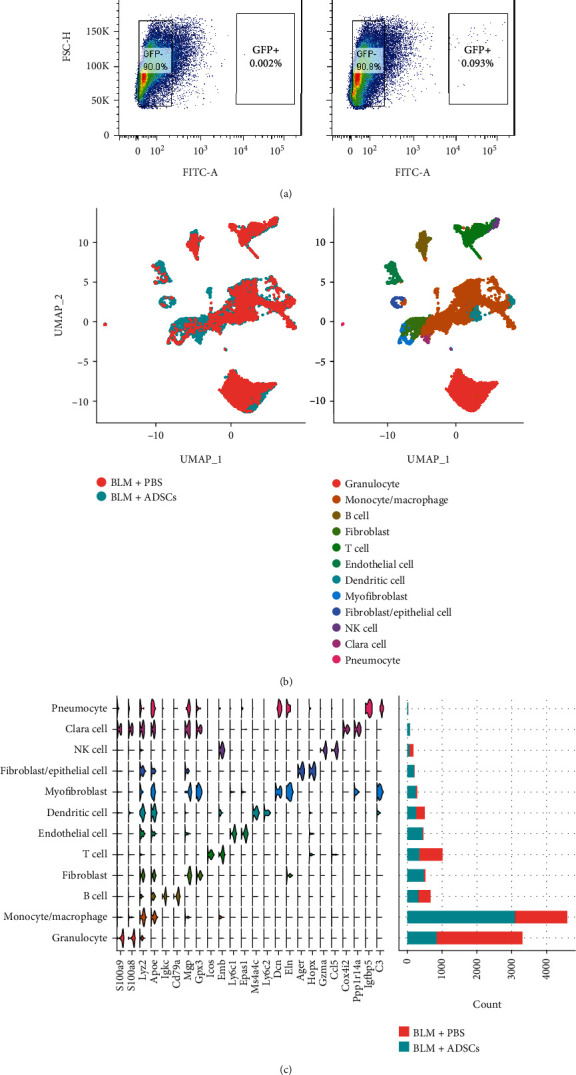
Injected ADSCs greatly changes single-cell heterogeneity of BLM-treated lung tissue. (a) Flow cytometry diagrams for sorting of digested lung tissue based on GFP signal intensity. Left: BLM + PBS group, as non-GFP control for lung-originated cells only. Right: BLM + ADSCs group, showing a small population of GFP+ cells, which are considered recollected ADSCs. (b) UMAP plot of lung-originated cell scRNA-seq data, merging cells from the BLM + ADSCs group and BLM + PBS group. Left: dots colored by group. Right: dots colored by SingleR annotated cell type based on clusters identified by Seurat. (c) Stacked violin plot of cluster marker genes (three marker genes for each cluster) and cell count number for each cluster of lung-originated cells.

To understand how ADSCs influence the PF environment, we sorted the GFP^+^ and GFP^−^ cells from the BLM + ADSCs and BLM + PBS groups lung tissue cell suspensions (Figure 2(a)) and then compared single cell profiles of lung-originated (GFP^−^) cells between the BLM + PBS and BLM + ADSCs groups (Figure 2(b)), comprising 8181 and 6405 cells, respectively. A total of 27 cell subgroups were identified in the merged dataset of lung-originated cells (Supplementary Figures 1(a)and 1(b)), and we identified 12 major cell types for each group of lung cells using SingleR (Figures 2(b) and 2(c)).

Among the major cell types, granulocytes were the most abundant in the BLM+PBS group, while monocytes/macrophages were the dominant cell type in the BLM+ADSCs group (Figure 2(c)), indicating drastic inflammation-related changes.

**Figure 3:**
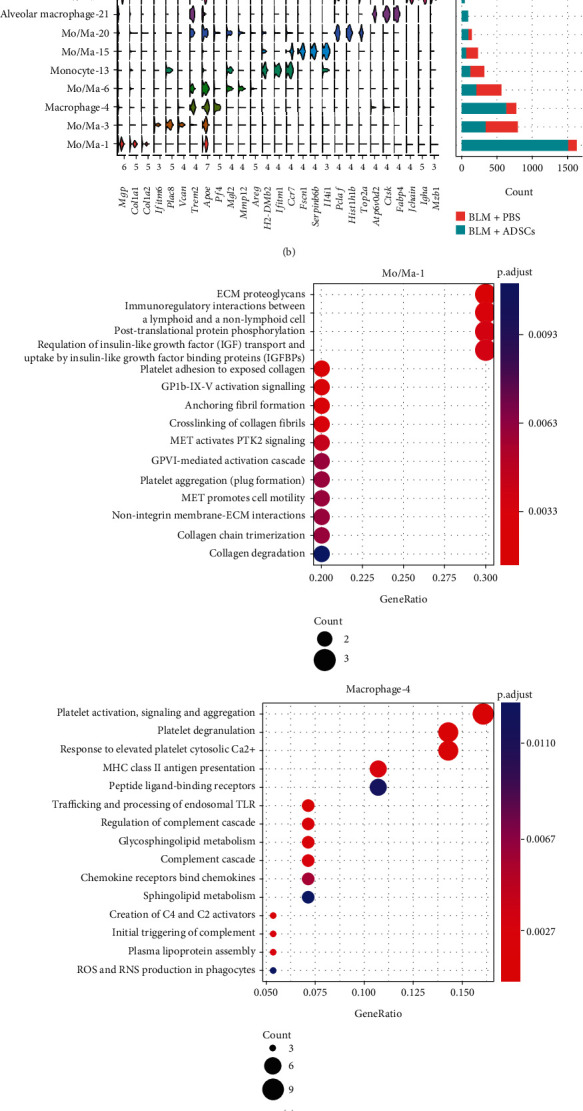
ADSC injection changed composition of macrophage population in BLM-treated lung. (a) UMAP distribution of 9 monocyte/macrophage clusters in lung-originated cells, merging the BLM + ADSCs group and BLM + PBS group. (b) Stacked violin plot of cluster marker genes (top three marker genes for each cluster) and cell count of monocyte/macrophage clusters. (c) REACTOME pathway analysis of marker genes of Mo/Ma-1 and macrophage-4 clusters. Genes are filtered by a condition of log2 fold change >0.5 and adjusted *P* value <0.1. (d) Immunofluorescence images of APOE and TREM2 which the highly expression genes in macrophage-4 on day 7 post BLM treatment.

Monocyte/macrophages contained 9 clusters with distinct transcriptomic profiles (Figures 3(a) and 3(b)). The Mo/Ma-1 cluster, characterized by marker genes *MPG*, *COL1a1*, and *SPARC*, was the most abundant population. The macrophage-4 cluster with high expression levels of *APOE* and *TREM2* was the second most abundant (Figure 3(b)). They were both predominantly composed of BLM + ADSCs cells, indicating that they were the potential target and/or effector of with ADSC intervention.

We next examined the function of genes with significant changes of expression levels in the Mo/Ma-1 and macrophage-4 subgroups based on REACTOME pathway analysis. Mo/Ma-1 cluster was related to the regulation of the ECM and proteoglycans, nonintegrin membrane-ECM interactions, collagen chain trimerization, collagen degradation, and crosslinking of collagen fibrils (Figure 3(c)). Macrophage-4 cluster showed higher activity in platelet activation, signaling, aggregation and degranulation, MHC II antigen presentation, and regulation of complement cascade. It was then determined as *Trem2*^+^ macrophages, whose abundance was confirmed to increase at day 7 in the BLM + ADSCs lung tissue, as indicated by the stronger fluorescence signals of TREM2 protein (Figure 3(d)). *TREM2* expression is positively linked to the severity of lung diseases such as viral infection [27] and chronic obstructive pulmonary disease [28], but it is believed to suppress pro-inflammatory responses [27, 29]. The macrophage-4 subgroup therefore might have alleviated the inflammation caused by BLM and ameliorated the subsequent fibrotic response.

### 3.3. ADSCs React to BLM-Treated Lung Environment with Dampened Metabolic Activity

**Figure 4:**
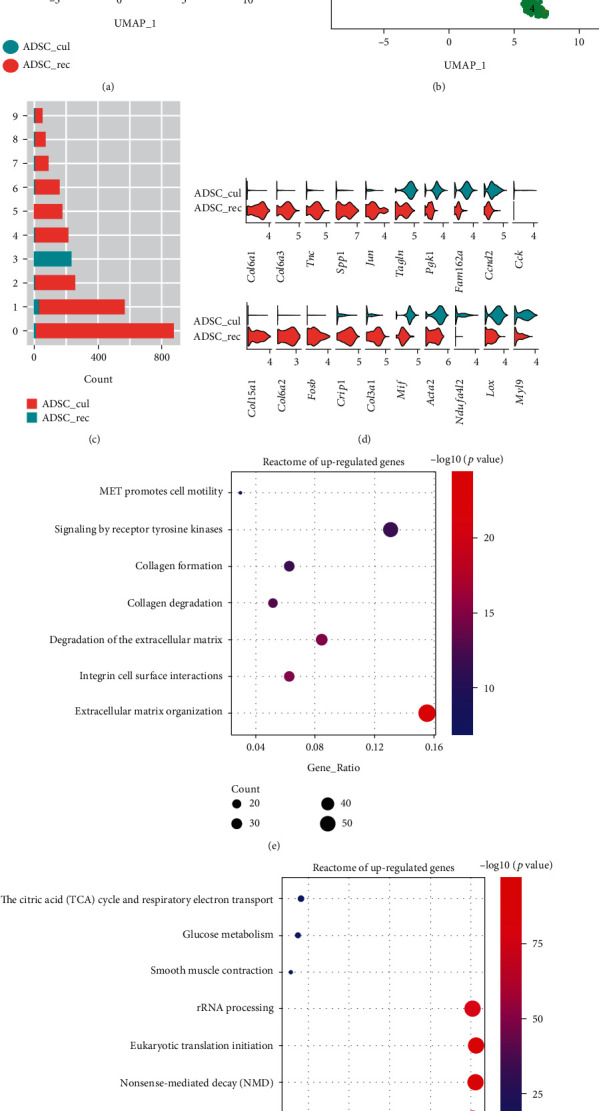
Recollected (ADSC_rec) ADSCs showed a different single-cell transcriptomic profile compared to cultured ADSCs (ADSC_cul). (a) UMAP distribution of in vitro cultured (ADSC_cul) and recollected (ADSC_rec) ADSCs merged into one dataset. (b) UMAP distribution of all 10 clusters identified by Seurat in merged dataset of ADSC_cul and ADSC_rec. (c) Cell count of every cluster in two ADSC groups. (d) Violin plots of differentially expressed genes between ADSC_cul and ADSC_rec groups. (e, f) REACTOME pathway enrichment terms of differentially expressed genes ((e) upregulated genes, (f) downregulated genes) in ADSC_rec group compared to ADSC_cul group. Genes were first filtered by a condition of log2 fold change >0.5 or < -0.5 and adjusted *P* value <0.05.

To investigate the potential therapeutic mechanism of intratracheally injected ADSCs in the BLM-treated inflammatory lung microenvironment (BLM + ADSCs group), we analyzed the single-cell transcriptomic profiles of recollected ADSCs from mouse lungs as well as ADSCs cultured in vitro. A total of 1 × 10^4^ GFP^+^ cells from three mice lungs (Figure 2(a)), namely, the recollected ADSCs (ADSC_rec), were sorted. Together with 5 × 10^4^ cultured ADSCs (ADSC_cul), they were subjected to scRNA-seq, with libraries consisting of 417 cells and 2801 cells, respectively. Unlike the ADSC_cul group, the ADSC_rec group does not show much heterogeneity, nor were they similar to any subgroup of ADSC_cul cells (Figures 4(a)–4(c)), which was likely the result of inadaptability of most cultured ADSCs upon contact with the inflammatory environment in BLM-treated lung. Notably, the ADSC_rec group showed high expression levels of ECM-related genes like *COL6a1/2/3*, *TNC*, and *COL3a1*, which were hardly expressed by ADSC_cul cells (Figure 4(d)). We further analyzed the functions of the genes with significant differences between ADSC_rec and ADSC_cul (log2 fold change >0.5 or < -0.5 and adjusted *P* value <0.05) using REACTOME pathway analysis. The upregulated genes of the recollected ADSCs were enriched to REACTOME pathway terms related to extracellular matrix remodeling and cell motility (Figure 4(e)), while their metabolic activity were downregulated as suggested by REACTOME enrichment, including glucose metabolism and RNA processing. (Figure 4(f)).

Recollected ADSCs showed elevated expression of ECM-related genes, while interestingly, histological assessments suggested that ADSCs decreased fibrotic ECM deposition in the lung tissue. It is not likely that injected ADSCs, which only take up a small proportion of ADSC-treated lung cells, was the major contributor of lung ECM reconstruction. And this small population of ADSCs was possibly adhering and adapting to the lung tissue through elevated ECM production compared to their cultured state *in vitro*. In the meantime, the low recollection rate can be explained by the inhibited metabolic activity compared to their cultured counterpart and is another proof that the majority of injected ADSCs cannot permanently reside in the pulmonary tissue, which is unfavorable for the claim that ADSCs transdifferentiate into functional pulmonary cells. Also, the characteristics of recollected subpopulation may provide a hint for improving ADSC survival after injection.

### 3.4. ADSCs Crosstalk with Lung-Originated Cells, Especially the *Trem2*^+^ Macrophages

**Figure 5:**
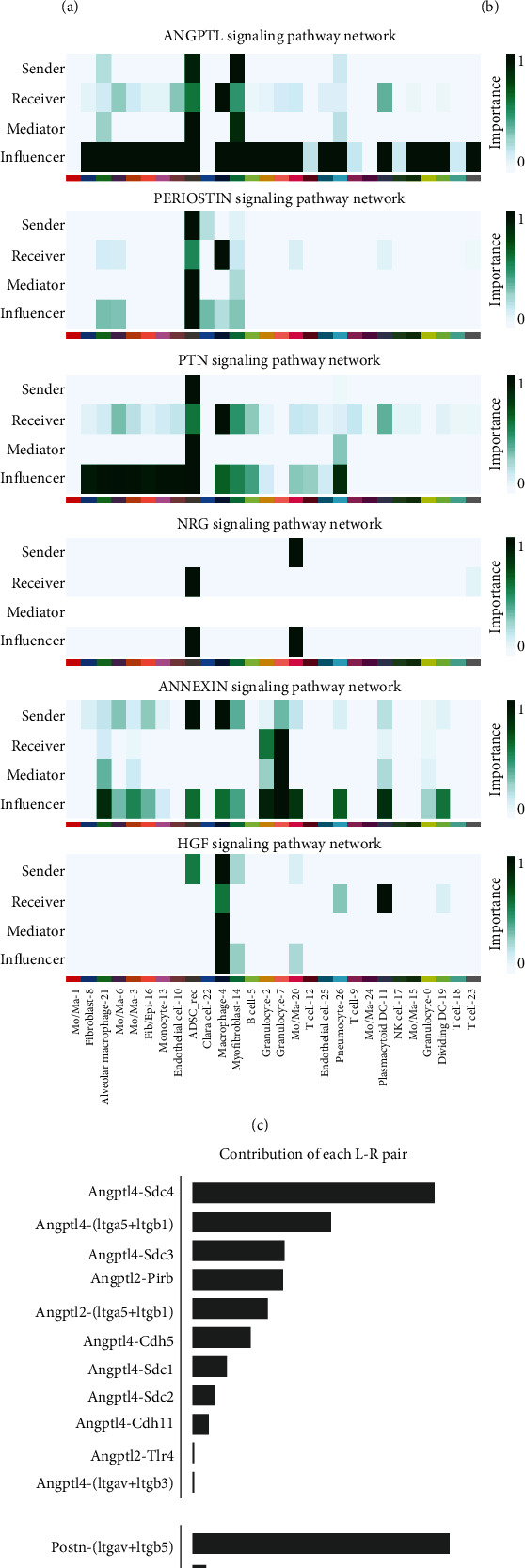
CellChat inference of signaling pathways among BLM + ADSCs group. (a) UMAP distribution of coarsely annotated cell type of both lung-originated cells and with recollected ADSCs (ADSC_rec) in BLM + ADSCs group. (b) Stacked violin plot of Ptprpc (CD45), CD68, and Egfp expression in BLM + ADSCs group. (c, d) Signaling pathway network in BLM + ADSC group calculated by CellChat. showing signaling between recollected ADSCs (ADSC_rec) and lung-originated cells. (c) Sender, receiver, mediator, and influencer of different pathways. (d) Relative contribution of each ligand-receptor pair in corresponding pathway network.

Due to the loss of most injected ADSCs, it is possible that they were gradually engulfed by recipient macrophages. To exclude this complication, we confirmed that the recollected ADSCs do not express *Ptprc* (CD45) or *Cd68* and was the sole subgroup that highly expressed *Egfp* (Figures 5(a) and 5(b)).

To investigate the communication between the injected ADSCs and the lung-originated cells in the BLM-treated lung, we identified the cell-cell interaction with CellChat. The recollected ADSCs mainly received NRG signaling (NRG1-(ITGAV+ITGB3)) from Mo/Ma-20 subgroup (Figures 5(c) and 5(d)). The NRG1-(ITGAV+ITGB3) binding is reported to be essential for NRG1-ERBB signaling [30], which may enhance ADSC migration and resistance to apoptosis [31].

In the meantime, ADSCs affect multiple lung-originated cell subgroups via paracrine signals (Figures 5(c) and 5(d)). The main receiver of ANGPTL, PERIOSTIN, and PTN was macrophage-4, with ANGPTL4-SDC4, POSTN-(ITGAV+ITGB5), and PTN-NCL as the top or sole contributor of ligand-receptor pairs, respectively. ANGPTL4 secreted by MSCs inhibits macrophage proinflammatory polarization [32]. POSTN, however, though reported to promote viability, proliferation, and migration of implanted ADSCs in a hind limb ischemia mice model [33], is also a biomarker and promoter of patient idiopathic PF [34]. PTN pathway plays an important role in fetal lung development and promotes proliferation of type II alveolar cells [35], and PTN protein can induce monocyte-endothelial transdifferentiation [36], but as the top target subgroup of PTN pathway, macrophage-4 yet did not express known endothelial biomarkers such as *Cd34* and *Tie2*. It is possible that these macrophages were also receiving other signals which counteracted or dampened the transdifferentiation-inducing effect of PTN signal.

ADSCs and macrophage-4 interacted with granulucytes-2/7, plasmacytoid dendritic cell (DC)-11, and some monocyte/macrophage subgroups via ANXA1-FPR2 pathway while also affecting macrophage-4 and plasmacytoid DC-11 via HGF. Annexin-A1 (Anxa1) counteracts BLM-induced PF [37] and mediates antiinflammatory effects in granulocytic differentiation [38]. HGF has been well studied for its proregenerative role in PF, whose effects include inhibition of epithelial/endothelial cell apoptosis and epithelial-to-mesenchymal transition and facilitation of myofibroblast apoptosis [39]. The macrophage-4 subgroup was the major provider and receiver of HGF signal in ADSC-treated lungs. Macrophage-secreted HGF is reported to promote epithelial repair in Crohn's disease patients, and macrophages under the HGF signal will undergo transition into an anti-inflammatory phenotype in skeletal muscle regeneration [40]. Moreover, plasmocytic DC-11 subgroup is another strong receiver of the HGF signal, and DCs are found to be regulated by MSC-secreted HGF to be immunologically tolerated, which is supportive for alleviating acute lung injury [41]. In our case, these paracrine signals secreted by ADSCs may have played a proregenerative and antiinflammatory role in their encounter with cells in BLM-treated lung, increasing the abundance of *Trem2*^+^ macrophage-4 subgroup and further affecting other inflammation-related cell types including granulocytes and DCs, which orchestrated towards a less inflammatory environment.

Interestingly, our data also suggest that not everything that ADSCs secret may be beneficial for fibrosis resolution, such as POSTN. This could provide potential targets for future precise engineering of ADSCs to weaken their negative effects while enhancing positive ones. And though further explanations need to be made on how cell-cell communication between the injected ADSCs and the damaged lung contribute to mitigating PF, this study represents the first step towards the direct detection of injected cells and understanding their interaction with recipient cells in cell therapy.

## 4. Conclusions

This study is the first to recollect injected ADSCs in mouse PF model and apply scRNA-seq for analysis of interaction between ADSCs and lung-originated cell types. Intratracheally injected ADSCs can alleviate BLM-induced PF, but their number diminishes over time, likely due to dampened metabolic activity, which suggests that ADSCs did not contribute to PF alleviation through transdifferentiation into functional pulmonary cells. Although we only recovered a small fraction of injected ADSCs, and that they did not possess as much heterogeneity as expected, we successfully analyzed the ligand-receptor interactions likely going on in the BLM-treated lung. The remaining ADSCs which were affected by the local NRG signal, through the secretion of effectors like ANGPTL4, HGF, and ANXA1, might have altered macrophages phenotype and influenced DCs to diminish their proinflammatory response. The *Trem2*^+^ antiinflammatory macrophage-4 subgroup was significantly more abundant after ADSCs injection in BLM-treated lungs and was also a major target of multiple ADSC-secreted factors, which is likely the key mechanism through which ADSCs alleviated fibrosis. These results indicated that the injection of ADSCs reduced a proinflammative response via activating *Trem2*^+^ antiinflammatory macrophages in BLM-induced PF. Our work shows the prospect of utilizing scRNA-seq to achieve a perspective closer to the *in vivo* interaction events between injected stem cells and the various types of cells in the disease-challenged tissue, as well as a better understanding towards the mechanisms behind MSC's therapeutic effects.

## Data Availability

The scRNA-seq data used to support the findings of this study are available from the corresponding author upon request.

## Supplementary Materials

Supplementary Materials Supplementary Figure 1: identification of 27 clusters of lung-originated cells in both groups of mice.
