# Supplementary material for: Single-Cell RNA Sequencing Reveals the Interaction of Injected ADSCs with Lung-Originated Cells in Mouse Pulmonary Fibrosis
Source: Stem Cells Int. 2022 Apr 11;2022:9483166. doi: 10.1155/2022/9483166 (PMC9017459; doi:10.1155/2022/9483166)
Supplement: Supplementary Materials — Supplementary Figure 1: identification of 27 clusters of lung-originated cells in both groups of mice. [file 9483166.f1.pdf]

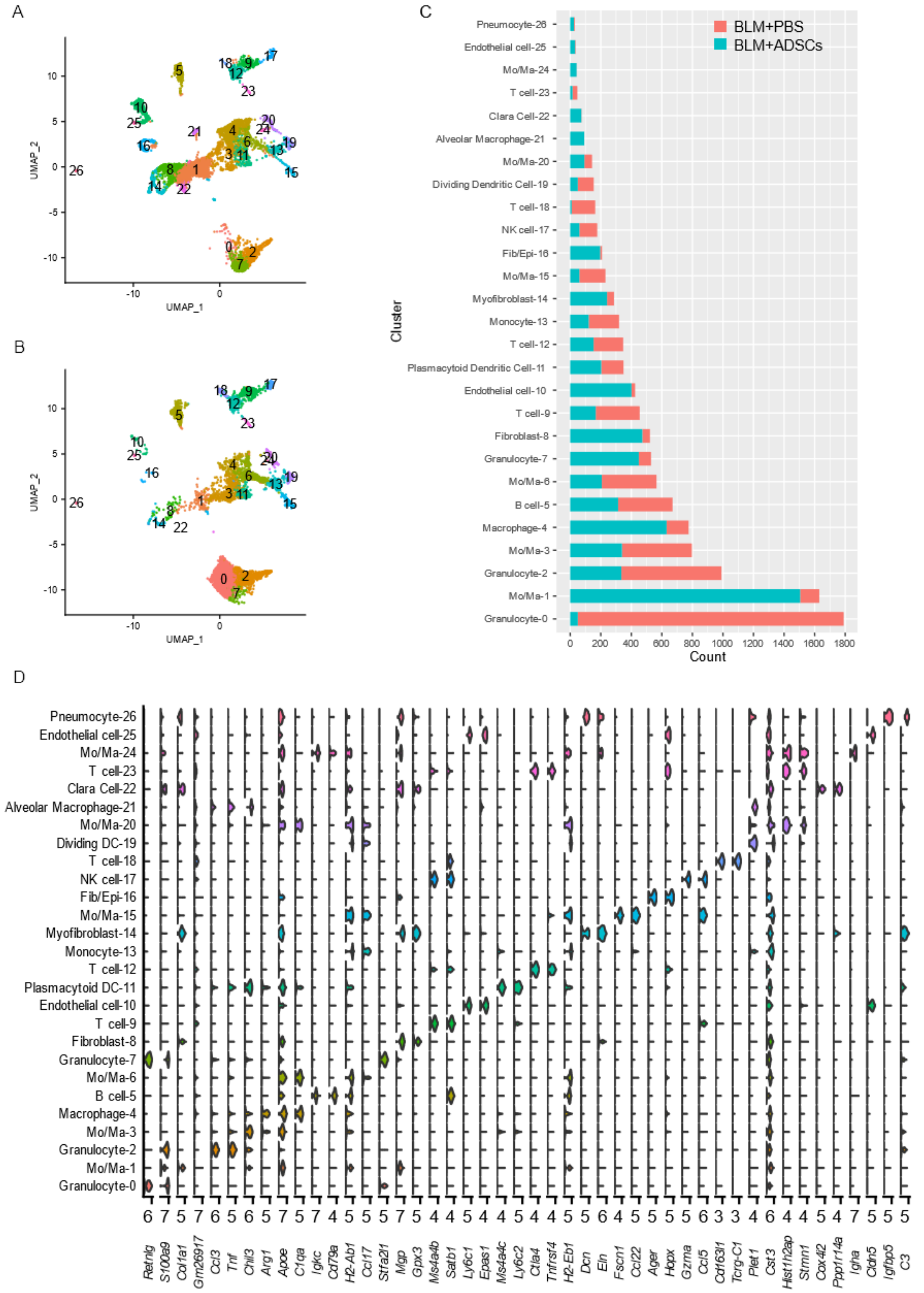

Supplementary Fig.1 Identification of 27 clusters of lung-originated cells in both groups of mice. **A,B**. UMAP distribution of all 27 clusters identified by Seurat in the merged dataset of BLM+ADSCs group (A) and BLM+PBS group (B). **C**. Cell counts in every cluster in each group. Cluster cell types were inferred by SingleR. **D**. Stacked violin plot of cluster marker genes (two marker genes for each cluster) and cell count number for each cluster of lung-originated cells.

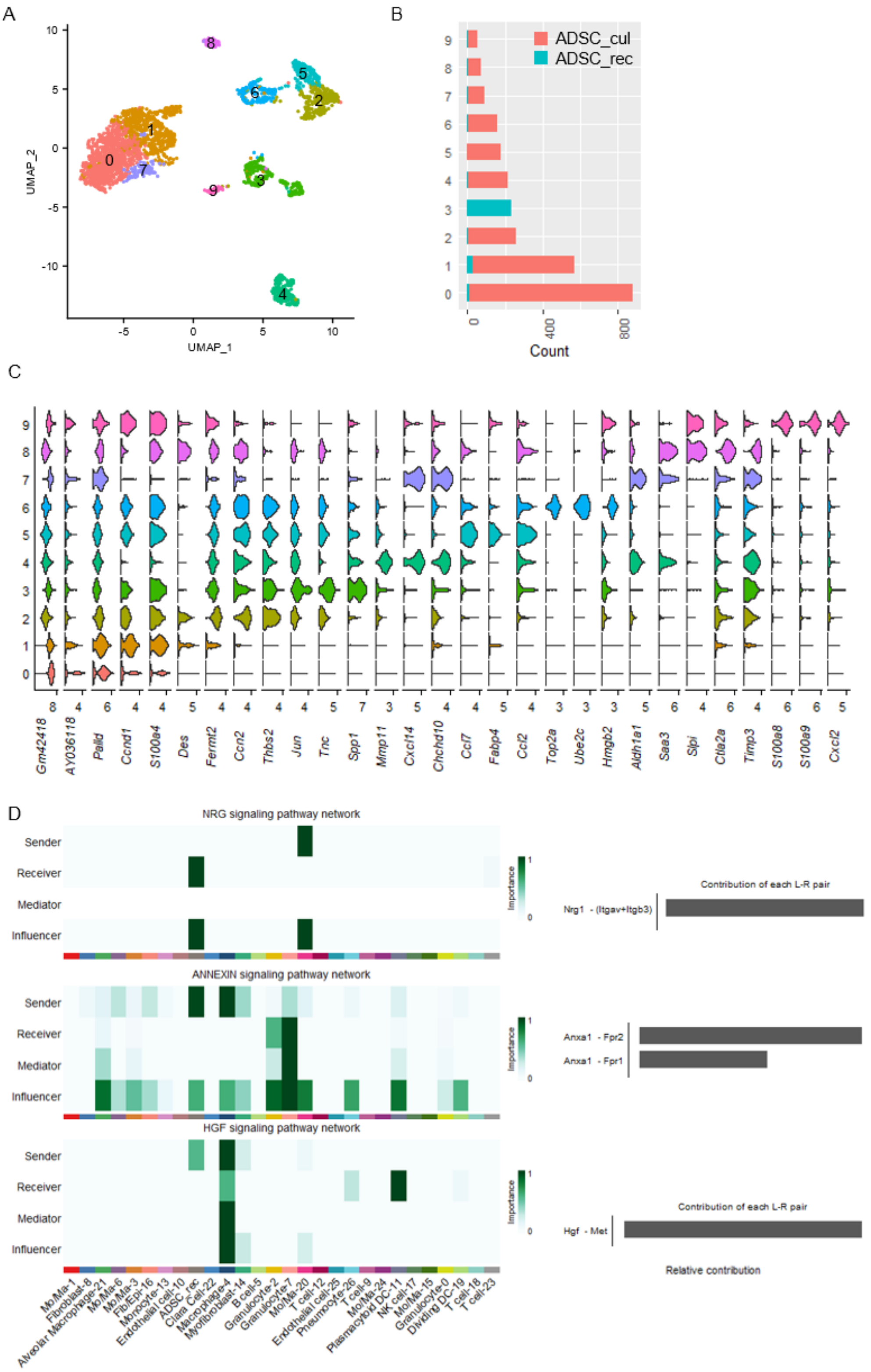

Supplementary Fig.2 Identification of 10 clusters of in-vitro cultured (ADSC_cul) and recollected ADSCs (ADSC_rec). **A**. UMAP distribution of all 10 clusters identified by Seurat in merged dataset of ADSC_cul and ADSC_rec. **B**. Cell count of every cluster in two ADSC groups. **C**. Stacked violin plot of cluster marker genes (top three marker

genes for each cluster) in two ADSC groups. **D**. Signaling pathway network in BLM+ADSC group calculated by CellChat, showing signaling with recollected ADSCs (ADSC_rec) as the receiver (NRG), and ADSC_rec as the sender when lung-originated cells other than Macrophage-4 was the major receiver. Left column: sender, receiver, mediator and influencer of different pathways. Right column: relative contribution of each ligand-receptor pair in corresponding pathway network.
